# Prevention strategies in stress coping and life crisis in trainees and early career psychiatrists

**DOI:** 10.1192/j.eurpsy.2025.260

**Published:** 2025-08-26

**Authors:** D. Gurrea Salas, ECP Task Force on Communication and Publications

**Affiliations:** Department of Addictive Disorders, Psychiatric Services Aargau (PDAG), Brugg, Switzerland

## Abstract

**Abstract:**

The current post-covid situation and the increase of violent situations in the ward within another risk factors flourished within the last years. With this input is aimed, the change of culture into the active implementation of stress and therefore mental health prevention strategies in the workplace and in safe spaces after the shift. Coping Strategies have to be implemented to improve the resilience and the ability to act (empowerment) in the early career stages of trainees and early career psychiatrists, as they are at risk of life crises and suicidal behaviors.

**Disclosure of Interest:**

None Declared

